# Tracing Geographical Origins of Teas Based on FT-NIR Spectroscopy: Introduction of Model Updating and Imbalanced Data Handling Approaches

**DOI:** 10.1155/2019/1537568

**Published:** 2019-01-03

**Authors:** Xue-Zhen Hong, Xian-Shu Fu, Zheng-Liang Wang, Li Zhang, Xiao-Ping Yu, Zi-Hong Ye

**Affiliations:** ^1^College of Quality & Safety Engineering, China Jiliang University, Xueyuan Street, Xiasha Higher Education District, Hangzhou 310018, China; ^2^BioCircuits Institute, University of California, La Jolla, San Diego, CA 92093, USA; ^3^Zhejiang Provincial Key Laboratory of Biometrology and Inspection & Quarantine, College of Life Sciences, China Jiliang University, Xueyuan Street, Xiasha Higher Education District, Hangzhou 310018, China; ^4^Department of Computer Science, Zhejiang University, Hangzhou 310027, China

## Abstract

This work presents a reliable approach to trace teas' geographical origins despite changes in teas caused by different harvest years. A total of 1447 tea samples collected from various areas in 2014 (660 samples) and 2015 (787 samples) were detected by FT-NIR. Seven classifiers trained on the 2014 dataset all succeeded to trace origins of samples collected in 2014; however, they all failed to predict origins for the 2015 samples due to different data distributions and imbalanced dataset. Three outlier detection based undersampling approaches—one-class SVM (OC-SVM), isolation forest and elliptic envelope—were then proposed; as a result, the highest macro average recall (MAR) for the 2015 dataset was improved from 56.86% to 73.95% (by SVM). A model updating approach was also applied, and the prediction MAR was significantly improved with increase in the updating rate. The best MAR (90.31%) was first achieved by the OC-SVM combined SVM classifier at a 50% rate.

## 1. Introduction

Tea is one of the most widely consumed beverages in the world because of its pleasurable taste, aroma, and healthy effects [1]. Freshly harvested tea leaves are processed differently to produce specific types of tea, such as unfermented green tea, fully fermented black tea and semifermented oolong tea. Of all the types, oolong has been proven to be able to reduce obesity and control diabetes [2].

Teas from different geographical origins vary in their quality due to different climate conditions (e.g., temperature, sun exposure time, and rainfall), soil, growth altitude, and so on. These factors would affect chemical compositions [3] of teas and thus determine their special aroma and taste—eventually their market prices. Many countries have published relevant regulations such as geographical indication (GI) certification to protect valuable products originated from certain areas. For example, as a GI product, the famous Wuyi rock-essence (WY) tea only grows in certain areas of Wuyishan city, Fuijan province of China (GB/T 18745–2006) [4]. However, driven by profits, special local teas are often replaced by inferior teas from other origins in the tea market. Therefore, it is of significant importance to develop reliable geographical origin tracing approaches for teas.

Among numerous techniques employed for origin tracing in the last decade, near-infrared (NIR) spectroscopy combined with pattern recognition methods has proved to be a valuable tool. Based on vibrational responses of chemical bonds to NIR radiation, this technique could provide different spectral fingerprints for products from different geographical origins. Many researchers have successfully applied NIR for origin tracing of honeys [5], olive oils [6], nuts [7], fruits [8], wines [9], wheats [10], herbal medicines [11], teas [12], and so on. In the case of teas, research studies can be subdivided into identifying teas from different nations [13], different areas within one nation [14], and different locations within the same geographical origin [15].

However, there exist some problems: First, in most cases, 60% to 75% of data from each group were selected for training. This kind of data-splitting strategy tends to raise the risk of over-fitting especially when each group consists of replicate samples; therefore, classifiers built based on this splitting strategy often give over-optimistic performances but would probably fail to predict data with different distributions.

Second, apart from the origin factor, chemical composition of tea leaves also varies depending on the “time” factor, such as harvesting timing, storage time, etc. [16]. In fact, NIR has also been reported to successfully discriminate teas with different storage periods [17] and harvest timing [18]. This raises the following question: how do the time factor and its interaction with the origin factor affect teas' NIR fingerprints for geographical traceability? Or is it possible to build a reliable NIR-based origin tracing model, despite changes in teas caused by other factors?

As far as we know, few research studies have been reported on this issue. Though there was a similar research on wheat, the result was not satisfying: Zhao et al. [19] tried to discriminate origins of wheats harvested from 2007/2008 and 2008/2009 using NIR; as a result, tracing models trained by data from one certain year could successfully predict samples from the same year, but totally failed to predict samples from the other year (only 39.2% accuracy).

In this research, a rapid and reliable geographical origin tracing approach based on Fourier transform near-infrared (FT-NIR) spectroscopy was proposed. A total of 1447 oolong tea samples from various origins were collected in 2014 and 2015 for the experiment. Seven classification algorithms were employed, and origin tracing models built based on them were compared. Three undersampling techniques as well as a model updating approach were also proposed to improve reliability of these models. The main objectives of this research were (1) to explore how the geographical origin factor, harvest time factor, and their interaction would affect teas' NIR fingerprints for geographical traceability; (2) to find solutions for classification tasks involved with imbalanced dataset and different data distributions; and (3) to build a reliable and robust origin tracing model for teas despite the changes in teas due to different harvest years.

## 2. Materials and Methods

### 2.1. Sample Preparation

The famous WY tea was selected as a representative of oolong for the geographical origin tracing research. To train a practical origin tracing model and avoid over-fitting, we tried to include as much diversity within each group as possible, i.e., tea samples for each origin (group) were collected from various areas (tea gardens) instead of just a few areas (with multiple replicates).

The whole research includes a two-year experiment. In 2014, 660 tea samples were collected to build a fast origin tracing model. Out of the 660 samples, 495 were authentic WY samples collected from 33 areas within the protected geographical origin, and 165 were Non-WY (NWY) oolong teas collected from 11 areas outside of the protected origin. In 2015, 787 tea samples were collected to validate if the origin tracing model trained by data from 2014 was still reliable and practical for tea samples from 2015. Out of the 787 samples, 687 were authentic WY samples collected from 74 areas within the protected origin, and 100 were NWY samples collected from 10 areas outside of the protected origin.

All the samples were spring teas (purchased before June) and were preserved in cold storage (4°C) before measuring. A description of the four tea groups was presented in Table 1.

### 2.2. NIR Sampling Procedure

A TENSOR37 FT-NIR spectrometer (Bruker, Ettlingen, Germany) was employed for the research. Each tea sample was packed in a quartz cuvette and detected with a PbS detector. Average values of 64 scanning spectra ranging from 4000 cm^−1^ to 12000 cm^−1^ were considered as the raw NIR data. Since the resolution was set as 8 cm^−1^ and the scanning interval was set as 1.928 cm^−1^, there were in total 4148 data points in the raw spectrum for each tea sample. Thus, the size of the raw dataset (2014 and 2015) was 1447 samples × 4148 variables. All the measurements were carried out at a room temperature of 25 ± 1°C.

### 2.3. Statistical Analysis and Pattern Recognition Methods

#### 2.3.1. Supervised Classifiers

Many classifiers have been widely applied for the analysis of NIR data. However, there exists no classifier that would always outperform others in every case. Thus, it would be better to try several common classifiers. In this paper, seven classifiers including both parameter and nonparameter approaches were applied to build geographical origin tracing models, and performances of these classifiers were compared. The classifiers employed here were as follows: linear discriminant analysis (LDA), support vector machine (SVM), stochastic gradient descent- (SGD-) based classifier, decision tree (DT), adaptive boosting- (AdaBoost-) based classifier, random forest (RF), and multilayer perceptron (MLP).

Developed by Fisher, LDA is a supervised approach that constructs discriminant functions through linear combination of labeled data. LDA consists of two stages: separation and allocation. The former stage is to find discriminant functions that can separate the groups well, and the later stage is to assign an unknown object to one of the groups using the discriminant functions.

SVM, which has been one of the most successful machine learning technique for the past decade, is a machine working in the high dimensional feature space formed by mapping of *n*-dimensional input vector into a *K*-dimensional feature space (*K* > *n*) through the use of a kernel function. The kernel function used here was radial basis function (RBF), which is able to reduce the computational complexity of the training procedure and to give a good performance under general smoothness assumptions [20]. The optimum parameter combination of gamma and *C* were found to be gamma = 0.001, *C* = 1.

SGD, which has been widely employed in solving large-scale machine learning problems, is a simple yet very efficient approach to discriminative learning of classifiers under convex loss functions such as linear SVM and logistic regression [21]. The loss function used here was linear SVM.

DT, which is a nonparametric learning method, predicts the value of a target variable by learning simple decision rules inferred from the data features. There are two common issues for the construction of decision trees: (a) the growth of the tree to enable it to accurately categorize the training dataset and (b) the pruning stage, whereby superfluous nodes and branches are removed in order to improve classification accuracy [22].

An AdaBoost classifier begins by fitting a classifier on the original dataset and then fits additional copies of the classifier on the same dataset but where the weights of incorrectly classified instances are adjusted [23]. In this paper, the base estimator from which the boosted ensemble was built was DTs, and the boosting algorithm used was the SAMME.R [24].

RF is a metaestimator that fits a number of DT classifiers on various subsamples of the dataset and use averaging to improve the predictive accuracy and control over-fitting. Each tree gives a classification, and the majority vote of trees in the forest is used to determine the final class [25]. In this research, the number of trees in the forest was set as 15.

MLP is composed of one input layer with *ρ* inputs, one or more hidden layers with *n* hidden neurons, and one output layer with *q* outputs. Selection of the optimum number of hidden layers as well as neurons in the hidden layers is important but also complicated. If an inadequate number of neurons are used, the network will be unable to model complex data, and the resulting fit will be poor. If too many neurons are used, the training time may become excessively long, and, worse, the network may over fit the data. The MLP employed here contained one hidden layer with 15 neurons, and error back propagation learning algorithm based on the Levenberg–Marquardt algorithm was used to train the neural network.

#### 2.3.2. Outlier Detection Based Undersampling Approaches for Imbalanced Classification

As described before, the WY and NWY classes were not represented equally: the ratio of 2014IN to 2014OUT was 695 to 165 (that is, 3 : 1), and that of 2015IN to 2015OUT was 687 to 100 (that is, 6.87 : 1). Imbalanced data like this could cause frustration because classifiers tend to favor the larger class, e.g., the classifiers might classify most of the tea samples as WY teas. Thus, it is important to balance classes in the training data.

The main objective of balancing classes is to either increasing the frequency of the minority class (over-sampling) or decreasing the frequency of the majority class (undersampling). In this research, three outlier detection based undersampling approaches—one-class SVM (OC-SVM), isolation forest (IF), and elliptic envelope (EE)—were proposed, and contamination of the lager class was set (among 0.38 to 0.55) with the goal of balancing the size of the WY and NWY classes.

Developed by Schölkopf et al. [26], the OC-SVM tries to find a hyper sphere which has maximal distance from the origin in feature space *F* and separates all the data points from the origin. This results in a binary function which captures regions in the input space where the probability density of the data lives. In general, the OC-SVM gives useful results in situations such as outlier detection in high dimension, or without any assumptions on the distribution of the inlying data.

IF builds an ensemble of trees for a given dataset; then anomalies are those instances which have short average path lengths on the trees [27]. This approach utilizes no distance or density measures to detect anomalies, which eliminates major computational cost of distance calculation in all distance-based and density-based methods.

EE is a common outlier detection approach that assumes data come from a known distribution (e.g., Gaussian distributed). From this assumption, this approach fits a robust covariance estimate to the data, and thus fits an ellipse to the central data points, ignoring points outside the central mode.

#### 2.3.3. Performance Measures for Imbalanced Classification

Accuracy is a popular performance measure index for classification tasks. However, as mentioned before, datasets for this research were not balanced. Thus, even if we build a completely useless classifier that classifies all the samples from 2015 as WY teas, we could still get an accuracy of 87.3% (687/787).

In such cases, recall and macro average recall (MAR) are more important. The recall for a class is the number of true positives (i.e., the number of items correctly labeled as belonging to the positive class) divided by the total number of elements that actually belong to the positive class (i.e., the sum of true positives and false negatives), and the MAR is the average of recall values for all classes (gives equal weight to each class).

#### 2.3.4. Model Updating

For a model to predict accurately, the data which are making predictions on must have a similar distribution as the data on which the model was trained. Thus, it is often a good practice to continuously monitor the incoming data and retrain the model on newer data when the new data distribution has deviated significantly from the original training data distribution.

In this research, because data distributions of the 2014 and 2015 groups were different, a model updating approach with different updating ratios—10% to 60%—was applied. During the model updating process, the 2015 dataset was randomly divided into 10 parts: part *X* was added to retrain the model, and the last four parts (parts 7–10) were considered as the testing set. *X* here could be any positive integers in the range of 1 to 6, representing the updating rate of 10% to 60%. This random-split process was repeated 100 times, and the average testing results were recorded for later analysis.

#### 2.3.5. Other Approaches Employed and Software Implemented

Principle component analysis (PCA) was employed to extract features from the original variables. One-way and two-ways analysis of variance (ANOVA) were performed to determine if there were differences among different groups, and Tukey's multiple comparison was performed to separate the means at *P* < 0.05.

All data analysis procedures were performed using Python 3, mostly the scikit-learn tool, which is a simple and efficient tool for data mining and data analysis.

## 3. Results and Discussion

### 3.1. NIR Responses to the Four Tea Groups

**Figure 1:**
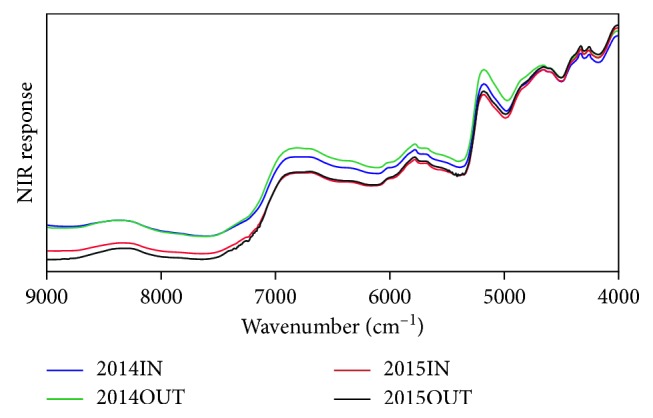
NIR responses (average values) to the four tea groups collected from different geographical origins in different years.

NIR responses (average values) to the four tea groups collected from different geographical origins and harvest years (marked as 2014IN, 2014OUT, 2015IN, and 2015OUT) are presented in Figure 1, where the *x*-axis represents the spectra scanning range, and the *y*-axis represents the NIR response. The wavenumbers from 9000 to 12000 cm^−1^ were excluded due to their lower sensitivity and signal-to-noise ratio [14].

As observed in Figure 1, there are some intensive spectral peaks in the region of 9000–4000 cm^−1^. These peaks were mainly generated by the stretch or deformation vibration of C-H, N-H, O-H, and C=O bonds, which are the primary structural components of organic molecules [28]. For example, the peak around 8330 cm^−1^ was induced by the second overtone of -C-H stretching, the peaks in 6000 to 7000 cm^−1^ were mainly induced by the O-H and N-H stretching, the peaks in 5500 to 6000 cm^−1^ were mainly induced by the fundamental stretching of -C-H, the -CH_2_, and the -CH_3_ overtone, the peaks around 5200 cm^−1^ were induced by the combination of O-H and C-O stretching, the peaks around 4700 cm^−1^ were induced by the combination of O-H bending and C-O stretching, and the peaks around 4300 cm^−1^ were induced by the combination of C-H stretching and -CH_2_ deformation. These vibrations were mainly caused by ingredients in teas such as catechins, polyphenols, alkaloids, volatile and nonvolatile acid, and some aroma compounds [29].

It is interesting to notice that the teas from the same harvest year (e.g., 2014IN and 2014OUT groups or 2015IN and 2015OUT groups) have much closer NIR responding curves than those from the same geographical origin (e.g., 2014IN and 2015IN groups or 2014OUT and 2015OUT groups) in the scanning range of 9000 to 5000 cm^−1^, suggesting the “harvest year” factor played a much bigger role than the “geographical origin” factor in that region. What is more interesting is, in the range of 9000 to 7250 cm^−1^, responding curves of the 2015IN and 2015OUT groups are separable while those of the 2014IN and 2014OUT groups are mostly overlapped; however, a totally reversed case is found in the range of 7250 to 5200 cm^−1^, where the responding curves of the 2015IN and 2015OUT groups are mostly overlapped while the 2014IN and 2014OUT groups are separable. Meanwhile, in the scanning range of 5000 to 4000 cm^−1^, the four groups seem to have relatively close responding curves. This does not seem great for our origin tracing tasks.

In summary, the NIR responses to teas were affected by both the “harvest year” and “geographical origin” factors, and the “harvest year” factor had a greater weight on most of the original spectral variables. This indicates that origin tracing models trained by data from one certain year might not be suitable for tea samples from the other year. Thus, it is important to eliminate the influence of the “harvest year” factor and find a feature subset that could better describe the differences in teas contributed by the “geographical origin” factor.

### 3.2. Preprocessing and Transformation of the Original NIR Data

PCA was applied for feature extraction. Meanwhile, since teas collected from different years have different data distribution centers (i.e., different group means), a standard scaling preprocessing approach was also employed to eliminate the influence of the “harvest year” factor, and data features from different years were all scaled to the [0, 1] range.

### 3.3. Discrimination of Teas Collected from Different Geographical Origins in 2014

The 660 tea samples from 2014 (495 in 2014IN and 165 in 2014OUT) were used to train and test origin classification models. Seven classifiers—LDA, SVM, SGD, DT, RF, AdaBoost, and MLP—were applied. To evaluate performances of these classifiers for teas collected from 2014, 70% of the dataset (462 sample) was randomly chosen as the training set, and the rest 30% was considered as the testing set. This random-split process was repeated 100 times, and the average training and testing results were recorded in Table 2, where recall 1 and recall 2 were recall values for the WY and the NWY groups, respectively.

As observed in Table 2, even though the LDA, SVM, DT, AdaBoost, and MLP classifiers were significantly better (alpha = 0.01) than the SGD and the RF classifiers in the case of the training set, all seven classifiers achieved high recall values with a minimum MAR value (equally weighted average of recall 1 and recall 2) of 98.95%. In the case of the testing set, all seven classifiers were able to discriminate the WY tea samples properly (recall 1 ≥ 91.32%); yet none of them could reach a recall2 value higher than 90%. This might be explained by the following two reasons: (1) the ratio of 2014IN to 2014OUT was 695 to 165; therefore, these classifiers tended to favor the larger group (WY group) by classifying more NWY samples as WY samples. (2) Data distributions of the 2014IN and 2014OUT groups were not far away from each other, and there might also be outliers around the potential decision boundaries, making it hard to draw clear decision boundaries that could fully discriminate the two groups. After calculating the prediction MAR for the testing set, the LDA and MLP classifiers were found to be the best (MAR ≥ 93%), followed by three equally good classifiers—the SVM (MAR = 89.65%), SGD (MAR = 88.51%), and AdaBoost (MAR = 88.27%) classifiers. Meanwhile, even though the DT classifier got the lowest MAR value (80.89%), it was still higher than 80%.

In general, all seven classifiers succeeded to predict geographical origins for the tea samples from 2014. This coincides with other researchers' works, where classifiers trained and tested by data from a one-time experiment always have good prediction accuracy. Now, our next question is, Are these origin tracing models reliable and robust? Or will they still be able to correctly classify tea samples from the next year?

### 3.4. Validation of the Origin Tracing Models Using the 2015 Tea Samples

To validate the aforementioned origin classification models, 787 new tea samples (687 WY teas and 100 NWY teas) were collected in 2015, and predictions of these new samples based on the seven classifiers were given in the last three columns of Table 2.

As observed in Table 2, the results are far from satisfactory. For all seven classifiers, there were significant descending in recall values: the prediction recall for the 687 new WY samples (recall 1) ranged from 58.42% to 90.74%, with the highest value achieved by the SVM (90.74%) and the lowest value provided by the MLP (58.42%); the prediction recall for the 100 new NWY samples was much worse—the recall2 ranged from 16.38% to 26.22%. In general, all seven classifiers failed to correctly predict the origins of the new tea samples from 2015. Even though the SVM classifier was significantly better than the other six classifiers at the 0.01 level, it only got a MAR of 56.86%, suggesting the origin tracing models trained by the 2014 dataset could not be directly applied to the 2015 dataset.

This phenomenon might be explained by the following two reasons: (1) as we mentioned before, the 2014 dataset was slightly imbalanced, and there were outliers around the decision boundary. Therefore, classifiers built based on the 2014 dataset tended to favor the WY tea group. (2) underlying data distributions of the 2014 and the 2015 datasets were not exactly the same. Compared to the 2014OUT group, data distribution of the 2015OUT group was closer to the decision boundaries. Therefore, most of data points from the 2015OUT group were distributed on the wrong side (2014IN group) of the already biased decision boundaries.

It is also worth noticing that the LDA and MLP classifiers, which were proven to be the best for the prediction of the 2014 dataset, are now significantly worse than the other approaches. This is because LDA usually generates good power with strong assumptions of multivariate normality of the explanatory variables and equal covariance among different populations; yet these assumptions are rare in practice. In this case, the discriminant functions that could separate the 2014 dataset well were not suitable for the 2015 dataset. As to the MLP, though it does not make any assumption regarding the underlying probability density functions or other probabilistic information about the pattern classes in comparison to other probability based models, it could easily get over-fitting especially when it was trained by a relatively small dataset.

### 3.5. Model Improving and Updating

In order to improve the origin identification models, three issues—imbalanced dataset, outliers, and different data distributions from the 2014 and 2015 datasets—must be solved.

#### 3.5.1. Dealing with Imbalanced Classification and Outliers

Three outlier detection based undersampling approaches—IF, OC-SVM, and EE—were employed to solve the issues of “imbalanced classification” and “outliers” in the 2014 dataset, and the results are presented in Table 3.

As observed in Table 3, no matter which of the three undersampling approaches was used, prediction MAR for the 2015 dataset based on the seven classifiers (especially SVM) were all improved. This improvement was achieved because of the significant increase in the prediction recall for the 100 NWY tea samples—the recall2 now ranges from 37% to as high as 76%, in contrast to the original range of 16.38% to 26.22%. However, it is noticeable that the prediction recall for the 687 WY tea samples was actually descending. This is in favor of our assumption that both the 2015IN and 2015OUT groups were closer to the decision boundaries than the 2014 groups. Therefore, when the issues of imbalanced dataset and outliers were taken care of, changes in new decision boundaries that favored the 2015OUT group would inevitably affect the 2015IN group.

In general, the OC-SVM and IF approaches, which are able to recover reasonable approximations even when the inlier distribution is strongly non-Gaussian, were better than the EE approach. Meanwhile, the highest prediction MAR for the 2015 dataset was improved from 56.86% to 73.95% by the SVM approach.

#### 3.5.2. Dealing with Different Data Distributions

A model updating approach with updating ratio of 10% to 60% was applied to further improve both the recall 1 and recall 2 for the tea samples from 2015. The idea of this updating approach is really simple: by adding some data from 2015 into the training process, the retrained classifier could find a more accurate discriminate boundary. Since the SVM classifier was better than the other classifiers in general, only the SVM classifiers combined with the OC-SVM and IF approaches were chosen for the model updating analysis.

**Figure 2:**
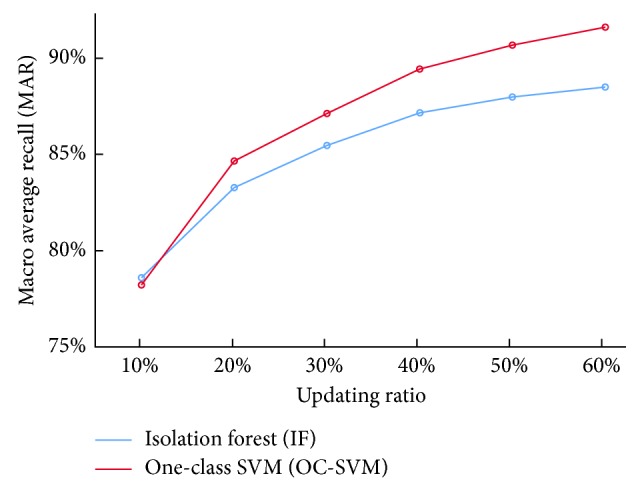
Prediction of tea samples from 2015 based on OC-SVM and IF combined SVM classifiers at different updating ratios.

Figure 2 visually displays how the prediction MAR for the testing set changed at different updating ratios under the two undersampling approaches.

As observed in Figure 2, for both the IF and OC-SVM combined SVM classifiers, prediction MAR increased—but the increasing rate decreased—with increase in the updating ratio. In general, the OC-SVM was better than the IF except at the ratio of 10%, where the IF seemed to have a slightly higher MAR value. Two-way ANOVA and post hoc test were then applied to analyze the results, and the interaction effect between the undersampling approach and updating ratio was found significant at 0.01 level (Table 4).

As observed in Table 4, for both the IF and OC-SVM combined SVM classifiers, identification performance for the WY (recall 1) and NWY (recall 2) teas got better with increase in the updating ratio up to 50%—there was no significant difference between the updating rate of 50% and 60% at 0.01 level. The best prediction performance for the tea samples from 2015 was first achieved by the OC-SVM combined SVM classifier at an updating rate of 50% (MAR = 90.31%). Meanwhile, even though the IF seemed to have a slightly higher MAR value than the OC-SVM at 10%, there was no significant difference between these two undersampling approaches at this updating rate.

## 4. Conclusions

It could be concluded from this work that both the harvest time and geographical origin factors had significant effect on NIR fingerprints of teas, and the harvest time factor seemed to have a bigger weight. Meanwhile, classification algorithms tended to favor the larger group for imbalanced datasets. Thus, if applied directly without solving the issues of different data distributions, outliers and imbalanced datasets, the origin tracing models trained by tea samples from one certain year would fail to predict tea samples collected from the other years.

This work also demonstrates that OC-SVM is a good outlier detection approach, and the OC-SVM based undersampling approach could be a powerful tool for imbalanced classification. The proposed model updating and outlier detection based undersampling approaches make it possible to build a reliable and robust origin tracing model that could be applied for teas collected from different years.

In summary, data distributions can be expected to drift over time, deploying a model is not a one-time exercise but rather a continuous process. Inspired by this research, our next plan is to adjust this model for teas with different storage times on a monthly basis.

## Figures and Tables

**Table 1:** Description of tea samples.

Year	Group ID	Type	Group size	Tea gardens per group	Raw variables per sample
2014	2014IN	WY^a^	495	33	4148
	2014OUT	NWY^b^	165	11	4148

2015	2015IN	WY	687	74	4148
	2015OUT	NWY	100	10	4148

^a^WY: authentic Wuyi rock-essence tea with a protected geographical indication (GI); ^b^NWY: Non-Wuyi tea.

**Table 2:** Comparative classification of tea samples based on seven different classifiers.

Classifier	Training set (70% of 14 dataset)			Testing set (30% of 14 dataset)			15 dataset		
	Recall 1	Recall 2	MAR^a^	Recall 1	Recall 2	MAR	Recall 1	Recall 2	MAR
LDA	0.9994	0.9983	**0.9989 A**	0.9826	0.8925	**0.9376 A**	0.6199	0.2347	**0.4273 C**
SVM	1.0000	1.0000	**1.0000 A**	0.9639	0.8291	**0.8965 B**	0.9074	0.2298	**0.5686 A**
SGD^b^	0.9987	0.9804	**0.9895 C**	0.9740	0.7961	**0.8851 B**	0.6621	0.1638	**0.4130 C**
Decision tree	1.0000	1.0000	**1.0000 A**	0.9132	0.7046	**0.8089 D**	0.8461	0.2461	**0.5461 B**
Random forest	0.9996	0.9904	**0.9950 B**	0.9703	0.7046	**0.8374 C**	0.8723	0.2195	**0.5459 B**
AdaBoost^c^	1.0000	1.0000	**1.0000 A**	0.9691	0.7963	**0.8827 B**	0.8370	0.2622	**0.5496 B**
MLP^d^	1.0000	1.0000	**1.0000 A**	0.9670	0.8957	**0.9314 A**	0.5842	0.2576	**0.4209 C**

^a^MAR: macro average recall; ^b^SGD: stochastic gradient descent; ^c^AdaBoost: adaptive boosting; ^d^MLP: multilayer perceptron. Means with the same letter(s) are not significantly different at 0.01 level.

**Table 3:** Improving the prediction of 2015 tea samples using three outlier detection based undersampling approaches.

Classifier	One Class SVM (OC-SVM)			Isolation forest (IF)			Elliptic envelope (EE)		
	Recall 1	Recall 2	MAR^a^	Recall 1	Recall 2	MAR	Recall 1	Recall 2	MAR
LDA	0.4993	0.3700	**0.4346**	0.4643	0.4100	**0.4372**	0.4498	0.3900	**0.4199**
SVM	0.7409	0.7300	**0.7355**	0.7191	0.7600	**0.7395**	0.7118	0.7500	**0.7309**
SGD^b^	0.5997	0.5100	**0.5549**	0.5997	0.5100	**0.5549**	0.5662	0.5100	**0.5381**
Decision tree	0.6652	0.5700	**0.6176**	0.6958	0.5800	**0.6379**	0.6201	0.5400	**0.5800**
Random forest	0.7089	0.6300	**0.6694**	0.6812	0.6200	**0.6506**	0.6710	0.6100	**0.6405**
AdaBoost	0.6405	0.6800	**0.6602**	0.7322	0.5400	**0.6361**	0.6667	0.5600	**0.6133**
MLP^c^	0.6856	0.5200	**0.6028**	0.8020	0.3800	**0.5910**	0.7205	0.3800	**0.5503**

^a^MAR: macro average recall; ^b^SGD: stochastic gradient descent; ^c^MLP: multilayer perceptron.

**Table 4:** Comparison of prediction results for 2015 dataset based on OC-SVM and IF combined SVM classifiers at different updating ratios.

Updating rate	One-class SVM (OC-SVM)			Isolation forest (IF)		
	Recall 1	Recall 2	MAR^a^	Recall 1	Recall 2	MAR
10%	0.8275	0.7324	**0.7799 G**	0.8414	0.7257	**0.7835 G**
20%	0.8482	0.8386	**0.8434 E**	0.8617	0.7983	**0.8300 F**
30%	0.8612	0.8749	**0.8680 D**	0.8717	0.8312	**0.8515 E**
40%	0.8739	0.9077	**0.8908 B**	0.8767	0.8599	**0.8683 D**
50%	0.8824	0.9238	**0.9031 A**	0.8802	0.8727	**0.8765 C,D**
60%	0.8867	0.9379	**0.9123 A**	0.8860	0.8770	**0.8815 B,C**

^a^MAR: macro average recall. Means with the same letter(s) are not significantly different at 0.01 level.

## Data Availability

The data used to support the findings of this study are available from the corresponding author upon request.
